# Exploring the associations between early maladaptive schemas and impulsive and compulsive buying tendencies

**DOI:** 10.3389/fpsyt.2023.1157710

**Published:** 2023-07-07

**Authors:** Susana Rocha, Xosé Manuel Fernández, Yolanda Rodríguez Castro, Simão Ferreira, Liliana Teixeira, Carlos Campos, Nuno Barbosa Rocha

**Affiliations:** ^1^Centre for Social and Organizational Studies (CEOS.PP), ISCAP - Porto Accounting and Business School, Polytechnic University of Porto, Porto, Portugal; ^2^Faculty of Education and Social Work, University of Vigo, Vigo, Spain; ^3^Center for Translational Health and Medical Biotechnology Research, School of Health, Polytechnic of Porto, Porto, Portugal; ^4^Center for Innovative Care and Health Technology, School of Health Sciences, Polytechnic of Leiria, Leiria, Portugal; ^5^Neurocognition Group | LabRP, Center for Rehabilitation Research, School of Health, Polytechnic Institute of Porto, Porto, Portugal; ^6^Digital Human‑Environment Interaction Lab (HEI‑LAB), Lusófona University, Porto, Portugal

**Keywords:** early maladaptive schemas, impulsive buying, compulsive buying, buying addiction, consumer behavior

## Abstract

The main purpose of this preliminary study was to investigate a potential relationship between early maladaptive schemas (EMSs) and impulsive and compulsive buying tendencies in a sample of young adults (college students). This research adds to the cognitive perspective of consumer behavior that the cognitive schemas putatively associated with early experiences may have a strong impact on impulsive and compulsive buying. Data was obtained from 365 participants in a cross-sectional study design. Participants completed an online survey with the following instruments: Young Schema Questionnaire; Impulsive Buying Tendency Measurement Scale; Richmond Compulsive Buying Scale; and Hospital Anxiety and Depression Scale. Using multiple linear hierarchical regressions, we confirmed that the domain of over vigilance and inhibition schemas was positively associated with impulsive and compulsive buying tendencies, while an opposite association was found for the domain of impaired limits. Being a female was also a predictor of impulsive buying and compulsive buying. The results were discussed in terms of the coping mechanisms to deal with negative emotions, as a way to obtain rewards, or as a way to escape painful self-awareness. Other mechanisms related to the internalization of perfectionist expectations and the propensity to shame were also explored.

## 1. Introduction

Impulsivity and compulsiveness are behavioral domains that are usually considered in the context of psychopathology. They are present in a wide range of mental disorders, such as obsessive–compulsive disorder, attention deficit hyperactivity disorder, autism spectrum disorders, addictions, among others (1). In general, impulsiveness is related to the devaluation of risk and obtaining immediate gratification. On the other hand, compulsiveness is related to learning a repetitive and maladaptive way of responding, with a low level of control in the presence of certain stimuli.

Impulsive buying is a product acquisition behavior that is done suddenly, immediately, and without planning or pre-purchase intention (2–4). The impulsive buying tendency is a buying pattern characterized by the propensity to feel spontaneous and sudden impulses to make purchases decided at the moment, based on the influence exerted by external stimuli, with reduced deliberation, cognitive control, and evaluation of the consequences (2). Usually, the post-purchase ends up degenerating into negative emotions and cognitive dissonance in which there is a general feeling of guilt for not being able to stop the impulse to buy.

When this way of buying becomes repetitive and causes negative consequences, such as financial, social, and psychological problems, it often degenerates into compulsive buying (5, 6). Existing evidence points to a 5 to 8% prevalence of compulsive buying in the general population, with other studies suggesting even higher numbers (7–13). This variation is explained by differences in the criteria used and in the target populations.

In general, compulsive acts relate to actions that are carried out in a persistent and repetitive manner, despite their adverse consequences (14). In the particular case of compulsive shopping, also called oniomania, there are three core elements: a maladaptive concern related to shopping, in which consumers feel an uncontrollable irrepressible and repetitive need to make purchases, even if they do not need those products; a noticeable loss of control over consuming behavior; and a continuation of excessive consumption, despite the negative consequences (5, 15, 16). In fact, the consequences are very negative for the person, who goes through the distress associated with the lack of control, the negative feelings that arise when they are not shopping, and the interference in their social, financial and occupational life (6, 17).

Although external factors are important for impulsive and compulsive buying, individual variables play a crucial role. Certain personality traits seem to be more related to the tendency for compulsive buying. Compulsive consumers usually show high extraversion (18), lower social cooperation and self-directedness (19), and increased neuroticism (20). Furthermore, there is strong evidence to support the idea that factors related to maladaptive beliefs serve as a background for compulsory shopping. Thoughts such as “if I shop will be more appreciated by others,” “shopping makes me feel that I am successful in my life” or “this product is unique and will help to improve my life” have an important impact on the etiology and maintenance of compulsive buying (17, 21, 22). These beliefs can be organized into the belief that the purchase of objects can compensate or neutralize negative emotions; emotional reasons to buy; the perception of objects as something unique; and the fear of losing good opportunities if the purchase is not made (22).

Within this cognitive perspective, we hypothesize that schema theory may offer a useful framework for compulsive buying. Young proposed a model that supported the development of therapy focused on schemas that constituted a significant development of the cognitive approach and that comes to integrate cognitive, emotional, relational, and behavioral variables (23, 24). Early cognitive schemas are the organizing construct of this conceptual model; they will allow a better understanding of the problems of individuals and enable the definition of therapeutic intervention strategies. According to Young, Klosko and Weishaar (25), p. 7 early maladaptive schemas are “broad, pervasive themes… regarding oneself and one’s relationships with others, which are developed during childhood or adolescence, elaborated throughout one’s lifetime, and dysfunctional to a significant degree.” These schemas result from core emotional needs that are not met and that lead to the perpetuation of dysfunctional patterns of thinking, decision-making, bodily sensations, behavior, and affect throughout life (26).

Currently, 18 major maladaptive schemas have been identified, organized into five domains: (1) Disconnection and rejection domain, which is related to the inability to create secure connections, based on the belief that the need for affection, love and belonging will not be met; (2) Impaired autonomy and performance domain, which is characterized by expectations about oneself and the environment that interfere with one’s perceived ability to function or perform successfully in an independently way, which are anchored in family functioning and overprotection and entanglement; (3) Impaired limits domain, which refers to serious difficulties in internal limits related to respecting others or achieving realistic personal goals, and which is believed to be associated with permissive and indulgent family functioning; (4) Other-directedness, which is defined by a constant and excessive cognitive focus on the approval by others, to the detriment of their own desires and feelings, which can be linked to a family pattern with relationships based on conditional approval; and (5) Overvigilance and inhibition, which is characterized by an excessive effort of self-control and suppression of feelings, as well as internalization of rigid patterns, possibly structured in a rigid and perfectionist family functioning.

We propose that individuals who have dysfunctional schemas are more likely to engage in compulsive and impulsive buying. We hypothesize that the overvigilance and inhibition maladaptive schema may underly excessive self-control and suppression of feelings, leading to rigid patterns and perfectionism, which are often associated with compulsive and impulsive buying. Compulsive and impulsive buying may be seen as a compensatory mechanism to alleviate negative situations or emotions, triggered by a need to overcome a negative self-perception (27, 28). Additionally, internalized perfectionist expectations (29–31) and materialistic values (32) are known to be associated with compulsive and impulsive buying. We believe that dysfunctional overvigilance and inhibition can lead to maladaptive self-approval and protection through impulsive and compulsive buying.

The main purpose of this preliminary study was to investigate a potential relationship between the EMSs and impulsive and compulsive buying in a sample of young adults (college students). In this study, we will rule out the role of adaptive cognitive schemas that could relate positively to impulsive or compulsive buying. This research adds to the cognitive perspective of consumer behavior, namely by discussing how cognitive schemas putatively associated with early experiences may have a strong impact on impulsive and compulsive buying.

## 2. Materials and methods

### 2.1. Participants

Four hundred and eighteen subjects completed the survey. Of these, 1 respondent was excluded for being aged below 18 years old; 6 who indicated they did not have Portuguese nationality, because there was no question in the survey regarding the degree of understanding of the Portuguese language; and 46 who did not respond “totally disagree” to the following control question: “I’m responding randomly to this survey.” The final sample comprised 365 participants, of which 36.4% were students in the area of health sciences, 55.6% were students of business sciences and 7.9% were students of engineering. The mean age was 22.41 years old and 72.1% were females. The demographic characteristics of the participants are shown in Supplementary Table S1.

### 2.2. Instruments

#### 2.2.1. The Young Schema Questionnaire – short form 3 SF3 (YSQ-S3)

The Young Schema Questionnaire – short form 3 (YSQ-S3) is a 90-item randomized version of the Young’s Schema Questionnaire assessing the 18 EMSs (33). Each item is rated using a six-point Likert scale, ranging from 1 = Entirely untrue of me to 6 = Describes me perfectly. For this study, we focused on five schema domains: Disconnection and Rejection; Impaired Autonomy and Performance; Impaired Limits; Other-Directedness; Overvigilance and Inhibition.

The only existing study of the Portuguese version of the scale confirmed the original factor structure and found good internal consistency, both for the total scale (*α* = 0.97) and for its subscales (between 0.571 and 0.861) (34). The internal consistency in our sample for the total scale was high (*α* = 0.96).

#### 2.2.2. Impulsive Buying Tendency Scale

The Impulsive Buying Tendency Scale (IBTS) (35) is a 20-item instrument comprising two facets: cognitive (IBTS-C) and affective (IBTS-A). The cognitive scale contains items related to the lack of planning and deliberation in purchasing decisions, and the affective scale addresses feelings of enthusiasm, lack of control, and urge to buy. Answers are given on a Likert scale ranging from 1 (Strongly disagree) to 7 (Strongly agree), with a higher score indicating a stronger tendency toward impulsiveness in purchasing. In the original study, the internal consistency values were *α* = 0.82 for the cognitive scale and *α* = 0.80 for the affective scale. In our sample, the internal consistency values were *α* = 0.88 for the cognitive scale and *α* = 0.81 for the affective scale.

#### 2.2.3. Richmond Scale for Compulsive Purchasing

The Richmond Scale for Compulsive Purchasing (RSCP) is an obsessive buying scale that was developed based on the rationale that compulsive is a disorder of the obsessive–compulsive spectrum, which includes a dimension of obsessive concern with the purchase and lack of control over the impulse to make a purchase (36). It consists of six questions, of which four are answered on a scale ranging from 1 (Strongly disagree) to 7 (Strongly agree) and two are answered on a scale ranging from 1 (Never) to 7 (Very often). A higher score is indicative of a greater compulsive buying tendency. The internal consistency for the full scale in the original study had a value of *α* = 0.84. In our sample, we obtained *α* = 0.82 of internal consistency.

#### 2.2.4. Hospital Anxiety and Depression Scale

Considering findings from Miltenberger et al. (37) that negative emotions, including anxiety and depression, are relevant antecedents of compulsive buying, we opted to control the effect of these variables on predictive models, using the Portuguese version of the Hospital Anxiety and Depression Scale (38). This instrument was developed to briefly assess the levels of depression and anxiety, and consists of 14 items, seven of which are for the assessment of anxiety (HADS-A) and seven for depression (HADS-D). Items are scored from zero to three, totaling a maximum score of 21 points for each subscale. The internal consistency of the Portuguese version for the two scales is good: *α* = 0.76 for the anxiety scale and *α* = 0.81 for the depression scale.

### 2.3. Procedures

The study was approved by the Ethics Committee of the ESS-P. PORTO. Participants were recruited from three schools from the North region of Portugal that gave authorization for the data collection. The participants answered the questionnaires in an online survey. They were informed about the purpose of the study and consented to participate in an online informed consent form written according to the Helsinki Declaration (39). Participants were not paid for their participation.

### 2.4. Data analysis

We conducted a hierarchical multivariate linear regression analysis with the tendency for impulsive and compulsive buying as dependent variables (impulsive buying – total score, impulsive buying – affective, impulsive buying – cognitive, and compulsive buying). For each model, block 1 included sociodemographic variables (age and sex), block 2 included anxiety and depression as state variables, and finally block 3 included the schema domains (Disconnection and Rejection, Impaired Autonomy and Performance, Impaired Limits, Other-Directedness, and Overvigilance and Inhibition).

Concerning the impulsive tendency variables, 16 participants’ z-score values were not between −3.29 and +3.29 in each analysis and were removed from the data. The remaining data (*n* = 349) showed that the Mahalanobis distances ranged between 0.808 and 29.113. In the case of compulsive buying, 21 participants’ *z*-score values were not between −3.29 and +3.29 in each analysis and were removed from the data. The remaining data (*n* = 344) showed that the Mahalanobis distances ranged between 0.798 and 29.404.

The critical value at the significance level of 0.001 for degrees of freedom 9 is 27.877. Thereby, two subjects with Mahalanobis distances higher than the critical value were excluded from the analysis. The final sample included in the impulsive buying models had 347 participants and in the compulsive buying model 342. Concerning multicollinearity, VIF values were lower than 10, and tolerance values higher than 0.20. The highest correlation between independent variables was 0.799 for impulsive buying models and 0.795 for the compulsive buying model.

We also checked the independence of residual assumptions, and the values of the Durbin–Watson statistic for the regression models for impulsive buying tendency ranged from 1.900 to 2.258, and for compulsive buying tendency the value was 0.297. The assumptions of linearity and homoscedasticity were verified by examining whether the residuals’ scatterplot resembles the shape of a rectangle and that the residuals were randomly scattered around the zero point and displayed a fairly even distribution. Finally, the normality assumptions were checked by observation of the normal probability plot, in which we confirmed that cases were distributed along a fairly straight diagonal line.

## 3. Results

### 3.1. Hierarchical linear regression models for impulsive buying tendency

The results regarding the regression model for Impulsive Buying Tendency Scale – Total Score (IBTS-TS) are presented in Table 1. The model for block 1 was significant, *F* (2, 346) = 3.280, *p* = 0.039, as well as the model for block 2, *F* (4, 346) = 3.162, *p* = 0.014. HADS – A was a significant predictor (*β* = 0.133, *p* = 0.031). In block 3 the model was significant *F* (9, 346) = 9.562, *p* < 0.001, and explained 20.3% of the variance on IBTS-TS. Sex (*β* = −0.124, *p* = 0.016), impaired limits (*β* = −0.225, *p* = 0.001) and overvigilance and inhibition (*β* = 0.627, *p* < 0.001) were significant predictors.

**Table 1:** Hierarchical linear regression analyses predicting the Impulsive Buying Tendency Scale – Total Score.

Predictors	Step 1		Step 2		Step 3	
	*β*	*t(p)*	*β*	*t(p)*	*β*	*t(p)*
Sex	−0.087	−1.599 (0.111)	−0.076	−1.399 (0.163)	−0.124	−2.425 (0.016)
Age	−0.090	−1.654 (0.099)	−0.076	−1.402 (0.162)	−0.065	−1.278 (0.202)
HADS anxiety			0.133	2.164 (0.031)	−0.006	−0.098 (0.922)
HADS depression			−0.004	−0.058 (0.954)	−0.064	−1.027 (0.305)
YSQ disconnection rejection					−0.045	−0.521 (0.603)
YSQ impaired autonomy performance					−0.063	−0.676 (0.500)
YSQ impaired limits					−0.225	−3.296 (0.001)
YSQ other-directedness					−0.030	−0.453 (0.651)
YSQ overvigilance and inhibition					0.627	6.537 (0.000)
*F* (df, df _error_)	*F* (2, 346) = 3.280, *p* = 0.039		*F* (4, 346) = 3.162, *p* = 0.014		*F* (9, 346) = 9.562, *p* = 0.000	
*R*	0.137		0.189		0.451	
*R* ^2^	0.019		0.036		0.203	
Δ*R*^2^	0.019		0.017		0.168	

The results for IBTS-A are presented in Table 2. The model for block 1 was significant, *F* (2, 346) = 5.369, *p* = 0.005, and age was a significant predictor (*β* = −0.121, *p* = 0.026). The model for block 2 was also significant, *F* (4, 346) = 6.718, *p* < 0.001, and HADS-A was a significant predictor (*β* = 0.219, *p* < 0.001). In block 3 (schema domains), there were significant changes in *R*^2^, *F* (9, 346) = 9.796, *p* < 0.001, which explained 20.7% of the variance on IBTS-A. Sex (*β* = −0.134, *p* = 0.009) and overvigilance and inhibition (*β* = 0.611, *p* < 0.001) were significant predictors.

**Table 2:** Hierarchical linear regression analyses predicting the Impulsive Buying Tendency Scale – Affective Domain.

Predictors	Step 1		Step 2		Step 3	
	*β*	*t(p)*	*β*	*t(p)*	*β*	*t(p)*
Sex	−0.103	−1.910 (0.057)	−0.086	−1.606 (0.109)	−0.134	−2.625 (0.009)
Age	−0.121	−2.243 (0.026)	−0.100	−1.881 (0.061)	−0.095	−1.876 (0.062)
HADS anxiety			0.219	3.626 (0.000)	0.083	1.333 (0.184)
HADS depression			−0.023	−0.385 (0.700)	−0.079	−1.283 (0.200)
YSQ disconnection rejection					−0.057	−0.669 (0.504)
YSQ impaired autonomy performance					−0.135	−1.449 (0.148)
YSQ impaired limits					−0.093	−1.358 (0.175)
YSQ other-directedness					−0.055	−0.832 (0.406)
YSQ overvigilance and inhibition					0.611	6.395 (0.000)
*F* (df, df _error_)	*F* (2, 346) = 5.369, *p* = 0.005		*F* (4, 346) = 6.718, *p* = 0.000		*F* (9, 346) = 9.796, *p* = 0.000	
*R*	0.174		0.270		0.455	
*R* ^2^	0.030		0.073		0.207	
Δ*R*^2^	0.030		0.043		0.135	

The results of the regression model for IBTS - C are presented in Table 3. Blocks 1 and 2 did not produce a significant model, *F* (2, 346) = 0.772, *p* = 0.463, and *F* (4, 346) = 0.450, *p* = 0.772. Block 3 produced a significant model, *F* (9, 346) = 6.133, *p* < 0.001, which explained 14.1% of IBTS - C. Impaired limits (*β* = −0.299, *p* < 0.001) and overvigilance and inhibition (*β* = 0.485, *p* < 0.001) were significant predictors.

**Table 3:** Hierarchical linear regression analyses predicting the Impulsive Buying Tendency Scale – Cognitive Domain.

Predictors	Step 1		Step 2		Step 3	
	*β*	*t(p)*	*β*	*t(p)*	*β*	*t(p)*
Sex	−0.049	−0.898 (0.370)	−0.048	−0.863 (0.389)	−0.083	−1.568 (0.118)
Age	−0.037	−0.669 (0.504)	−0.034	−0.606 (0.545)	−0.019	−0.361 (0.719)
HADS anxiety			0.016	0.251 (0.802)	−0.092	−1.423 (0.156)
HADS depression			0.017	0.267 (0.790)	−0.032	−0.501 (0.616)
YSQ disconnection rejection					−0.021	−0.238 (0.812)
YSQ impaired autonomy performance					0.023	0.240 (0.810)
YSQ impaired limits					−0.299	−4.218 (0.000)
YSQ other-directedness					0.002	0.031 (0.976)
YSQ overvigilance and inhibition					0.485	4.870 (0.000)
*F* (df, df _error_)	*F* (2, 346) = 0.772, *p* = 0.463		*F* (4, 346) = 0.450, *p* = 0.772		*F* (9, 346) = 6.133, *p* = 0.000	
*R*	0.067		0.072		0.375	
*R* ^2^	0.004		0.005		0.141	
Δ*R*^2^	0.004		0.001		0.136	

### 3.2. Hierarchical linear regression model for compulsive buying tendency

The results for the RSCP are presented in Table 4. The model related to block 1 was significant, *F* (2, 341) = 5.796, *p* = 0.003. Sex was a significant predictor (*β* = −0.154, *p* = 0.005). Block 2 also produced a significant model, *F* (4, 341) = 3.329, *p* = 0.011, and sex remained a significant predictor (*β* = −0.146, *p* = 0.008). On block 3 there were significant changes in *R*^2^, *F* (9, 341) = 7.552, *p* < 0.001, which explained 16.2% of the variance in RSCP. Sex (*β* = −0.194, *p* = 0.000), impaired limits (*β* = −0.182, *p* = 0.010), and overvigilance and inhibition (*β* = 0.468, *p* < 0.001) were significant predictors in this model.

**Table 4:** Hierarchical linear regression analyses predicting the Richmond Scale for Compulsive Purchasing.

Predictors	Step 1		Step 2		Step 3	
	*β*	*t(p)*	*β*	*t(p)*	*β*	*t(p)*
Sex	−0.154	−2.833 (0.005)	−0.146	−2.681 (0.008)	−0.194	−3.688 (0.000)
Age	−0.073	−1.341 (0.181)	−0.067	−1.230 (0.220)	−0.066	−1.262 (0.208)
HADS anxiety			0.078	1.267 (0.206)	−0.020	−0.321 (0.749)
HADS depression			−0.018	−0.301 (0.764)	−0.053	−0.836 (0.404)
YSQ disconnection rejection					−0.130	−1.475 (0.141)
YSQ impaired autonomy performance					−0.037	−0.386 (0.699)
YSQ impaired limits					−0.182	−2.592 (0.010)
YSQ other-directedness					0.109	1.588 (0.113)
YSQ overvigilance and inhibition					0.468	4.743 (0.000)
*F* (df, df _error_)	*F* (2, 341) = 5.796, *p* = 0.003		*F* (4, 341) = 3.329, *p* = 0.011		*F* (9, 341) = 7.552, *p* = 0.000	
*R*	0.182		0.195		0.402	
*R* ^2^	0.033		0.038		0.162	
Δ*R*^2^	0.033		0.005		0.124	

## 4. Discussion

To the best of our knowledge, this is the first study associating early maladaptive schemas with impulsive and compulsive buying tendencies. Overall, we confirm that early maladaptive schemas appear to play an important role in impulsive and compulsive shopping. Furthermore, this association was still significant despite broad domain psychopathology variables such as anxiety and depression.

Schemas are cognitive structures that develop over time in the interaction with the environment, which are installed in our autobiographical memory, and that explain how experiences that occurred in the past influence the processing of new information and its assimilation in the existing belief structure and, consequently, the way in which decisions are usually made (40, 41). In maladaptive schemas, one acts in a dysfunctional way, generating automatic dysfunctional thoughts, as well as unregulated emotional states, which can manifest themselves in different ways (26).

These can be an overwhelming or unregulated sadness, including the feeling of emotional emptiness, loneliness, and the feeling of not being loved; severe anguish, associated with an extreme fear of being abandoned; exaggerated shame; deregulated anger; impulsiveness and lack of control, with difficulty in postponing gratification and inability to predict the consequences of actions; and other dysfunctional emotional manifestations. At the same time, dysfunctional forms of coping can be activated (26, 42–44), such as avoiding situations, suppressing feelings, depersonalization, compulsive commitment to distracting and relief activities, breach of rules, acting without consideration for others, attack and bullying, ceaseless seeking for attention and approval, extravagant behavior, over-perfectionism, manipulation, among many others.

The application of the concept of maladaptive schemas is widely comprehensive and has been used to explain and predict results in conditions as diverse as personality traits and disorders (45–51), emotional regulation and attachment (52–55), suicide risk (56), sexual disorders (57–59), substance abuse (60, 61), and mental disorders (62–66).

Firstly, we found that the domain of the overvigilance and inhibition schema is the main predictor of both impulsive and compulsive buying. This finding that the same maladaptive schema domain is the main influence on both impulsive and compulsive buying reinforces the argument of relative overlap between impulsivity and compulsiveness. From a clinical point of view, it appears to be a relative overlap of endophenotypes in various disorders of the impulsive and compulsive spectrum (Impulsive Compulsive Spectrum Disorders), despite their different characteristics and the distinct manifestations of impulsiveness and compulsiveness. Impulsiveness is the propensity to respond without much thought or the inability to inhibit a response, while compulsion is repetitive, rigid, and perseverative behavior (67–72). This same relative overlap, which is obviously not complete, also seems to happen in the relationship between impulsive and compulsive buying (73, 74).

At first glance, our hypothesis proposing an association between overvigilance and inhibition schema with compulsive and impulsive buying may seem counterintuitive. It would be expected that excessive control of spontaneous impulses, avoidance of mistakes, and strict adherence to rules while being hypercritical would prevent these buying behaviors. However, there is evidence showing that people with a predominance of dysfunctional schemas tend to exhibit dysfunctional, immature and compensatory forms of coping (75). In addition, the continuous suppression of emotional expression can prepare a fertile ground for episodes of greater lack of control (76).

Thus, it is possible that the use of buying as a source of obtaining pleasure or without much reflection works as a compensatory mechanism in the case of excessive overvigilance and inhibition schemas. One possibility is that compulsive and impulsive buying behaviors work as a compensatory mechanism for these schemas, which can function as a coping strategy to bring some relief from negative situations or emotions or as a way to obtain satisfaction and reward, particularly triggered by a need to escape from a negative self-awareness (27, 28, 77, 78). In fact, there is evidence that people who shop compulsively have low self-esteem (79). Also, there are positive results from the use of antidepressants in the treatment of people with compulsive buying, which further highlight the potential role of negative emotions in these buying tendencies (80). According to Faber’s escape theory (2004), the involvement in immediate and concrete tasks, which is the case of buying, could help to escape from or compensate for painful self-awareness.

In this scenario, the action of these schemas would thus be paradoxical. By directing information processing toward the negative aspects of life and negative emotions and making the person afraid of a negative assessment by others, someone with these schemas would be more vulnerable to situations where obtaining rewards is more immediate or in which they understand that they can find an increased personal appreciation using external objects, as with shopping. Existing evidence shows that compulsive shoppers feel better and have a reduction in negative emotions after making a purchase (37, 81).

Another possible explanation comes from the existing evidence that impulsive and compulsive behaviors, as well as the obsessive–compulsive disorder itself, are often associated with the internalization of perfectionist expectations, the fear of making mistakes, increased responsibility, and high standards (29–31, 82–85). At the same time, a greater materialist appreciation for the signs of wealth and luxury (32), narcissistic traits (86–89), and perfectionism (21, 90) are present in impulsive and compulsive buying.

The cognitive schemas underlying the establishment of excessive patterns, self-depreciation, and high self-criticism are associated with forms of perfectionism (91–94) and even grandiose narcissism (95). Thus, it is possible that a mechanism exists in which the schemas that cause the suppression of positive impulses and the excessive inhibition of emotions (especially negative ones such as anger) contribute to a dysfunctional perfectionism that results in the adoption of behaviors that promote a maladaptive approval and protection of the self, which could be the case in impulsive and compulsive shopping, in addition to other external strategies for regulating negative emotions (e.g., alcohol abuse, overeating).

In addition to our main hypothesis, we have also found evidence of a negative association between impaired limits and impulsive/compulsive buying tendencies. Regarding impulsive buying tendency, we found that the deteriorated limits were associated with the cognitive domain, but not with the affective. The affective processes of impulsive shopping are related to an irresistible urge to buy, to the emotions of pleasure and excitement that one feels when buying, and to the possible guilt after buying. The cognitive domain of impulsive buying concerns whether the purchase is made in a thoughtful, planned, and deliberate way, whether it is only carried out according to needs and whether it is made with a comparison of alternatives (35).

The core beliefs associated with impaired limits are lack of responsibility, avoidance of discomfort, and feelings of superiority. These themes are apparently irreconcilable with the high levels of internalization of expectations and self-criticism that seem to promote impulsive and compulsive buying (21, 90). Thus, a certain degree of relaxation and distraction from responsibilities can provide some protection against compulsive buying, lack of planning and reflection typical of the cognitive dimension of impulsive buying.

From another perspective, the propensity to shame is an important dispositional risk factor for compulsive buying (96). Interestingly, the propensity for shame is associated with punitive and coercive parenting styles (97–99). The schemas’ impaired limits domain is often developed in indulgent and permissive family environments, which, despite being associated with several problems, can provide some protection against the propensity for shame and, consequently, against impulsive and compulsive buying. Despite these explanations, we cannot, however, rule out the possibility that this relationship in the opposite direction could be a multicollinearity statistical artifact.

Finally, sex was a significant variable explaining affective impulsive and compulsive buying, as being a women was a significant predictor in both models, as also indicated by previous evidence (15). Being a female has been found to be a predictor of the affective domain but not the cognitive domain of impulsive buying. This finding supports existing evidence that suggests females may display greater impulsivity due to factors such as being more easily compelled to buy with a strong emotional charge or being more attracted to hedonic purchases (27, 100, 101). Previous studies have examined gender differences in brand commitment, impulse buying, and hedonic consumption, indicating that women may be more prone to engaging in impulsive buying driven by affective factors, such as expressing love for someone close or seeking hedonic experiences (101).

Furthermore, research has identified specific factors contributing to female’s impulse buying tendencies. For instance, negative urgency and self-perceived attractiveness have been linked to female’s impulse buying behaviors (102). Several studies consistently demonstrate that female are more vulnerable to compulsive buying behavior compared to males. Females score higher on compulsive buying scales, indicating a greater susceptibility to using buying behaviors to regulate emotions and moods (103). Some studies even suggest that female compulsive buyers may resort to excessive buying as a way to cope with stress and negative emotions, while the pleasures and joy experienced in shopping may have a stronger impact on women than males (104, 105).

However, it is important to note that sex differences in compulsive buying are not universally consistent across all studies. Research conducted with adolescent and university student samples in Western countries failed to find significant sex differences in compulsive buying (106, 107). Additionally, a study with German undergraduate students even reported lower levels of compulsive buying among females compared to males (108).

Having this in mind, while females generally exhibit higher levels of brand commitment, hedonic consumption, and impulse buying compared to males, gender differences in compulsive buying behavior are not consistently observed across all studies. Cultural and contextual factors may play a role in shaping these gender differences, and the results presented in this paper reflect the Portuguese reality. Further research is needed to gain a better understanding of the complex interplay between gender, individual traits, and societal influences on buying behaviors.

This study has several limitations. Firstly, the cross-sectional design requires great caution when discussing causality mechanisms. Second, data collection was self-reported, which can increase the effect of social desirability on the responses. Third, the survey was an extensive online form, which may increase the risk of random responses, even though we were careful to insert a control question to reduce this limitation. Fourth, we used a sample of university students, and we had a predominance of female respondents.

## Data availability statement

The raw data supporting the conclusions of this article will be made available by the authors, without undue reservation.

## Ethics statement

The studies involving human participants were reviewed and approved by Porto’s School of Health - Polytechnic University of Porto Ethics Committee (ESS|P.PORTO). The patients/participants provided their written informed consent to participate in this study.

## Author contributions

SR: conceptualization. SR, XF, YC, SF, LT, and NR: methodology. SR, CC, SF, LT, and NR: data curation and analysis. XF and YC: supervision. SR, XF, YC, SF, LT, CC, and NR: original draft and review and editing. All authors contributed to the article and approved the submitted version.

## Funding

This work is financed by portuguese national funds through FCT - Fundação para a Ciência e Tecnologia, under the project UIDP/05422/2020.

## Conflict of interest

The authors declare that the research was conducted in the absence of any commercial or financial relationships that could be construed as a potential conflict of interest.

## Publisher’s note

All claims expressed in this article are solely those of the authors and do not necessarily represent those of their affiliated organizations, or those of the publisher, the editors and the reviewers. Any product that may be evaluated in this article, or claim that may be made by its manufacturer, is not guaranteed or endorsed by the publisher.

## Supplementary material

The Supplementary material for this article can be found online at: https://www.frontiersin.org/articles/10.3389/fpsyt.2023.1157710/full#supplementary-material
