# Supplementary material for: Exploring the associations between early maladaptive schemas and impulsive and compulsive buying tendencies
Source: Front Psychiatry. 2023 Jul 7;14:1157710. doi: 10.3389/fpsyt.2023.1157710 (PMC10362270; doi:10.3389/fpsyt.2023.1157710)
Supplement: Supplementary file 1 [file Table_1.docx]

Supplementary Material

Exploring the Associations between Early Maladaptive Schemas and Impulsive and Compulsive Buying Tendencies

Susana Aguiar Rocha^1,2^, Xose Manuel Cid Fernandez^1^, Yolanda Rodríguez Castro^1^, Simão Ferreira^3^, Liliana Teixeira^3^, Carlos Campos^4^, Nuno Barbosa Rocha^3^

^1^ Centre for Social and Organizational Studies (CEOS.PP), ISCAP - Porto Accounting and Business School, Polytechnic University of Porto (P.PORTO), Porto, Portugal

^2^ Faculty of Education and Social Work, University of Vigo, Viga, Spain

^3^ Center for Translational Health and Medical Biotechnology Research, School of Health, Polytechnic of Porto (P.PORTO), Porto, Portugal.

^4^ Digital Human-Environment Interaction Lab (HEI-Lab), Lusophone University, Lisbon, Portugal

*** Correspondence:**Dr. Susana Aguiar Rocha
srocha@iscap.ipp.pt

# Supplementary Table

***Table 1****. Demographics*

| **Variable** | **n(%) or M(SD)** |
| --- | --- |
| **Genre** |  |
| Male | 102 (27.95) |
| Female | 263 (72.15) |
| **Age** | 22.41 (4.42) |
| **Marital status** |  |
| Single | 347 (95.1%) |
| Married | 11 (3.0%) |
| Cohabiting | 4 (1.1%) |
| Divorced | 2 (0.5%) |
| Widowed | 1 (0.3%) |
| **Enrolled in a degree** |  |
| BSc | 349 (95.6%) |
| MSc | 16 (4.4%) |
| **Occupation** |  |
| Student | 267 (73.2%) |
| Student + part-time job | 61 (16.7%) |
| Student + full-time job | 37 (10.1%) |
| **Socio-economic level** |  |
| Low | 9 (2.5%) |
| Middle–low | 115 (31.5%) |
| Middle | 217 (59.5%) |
| Middle–high | 23 (6.3%) |
| High | 1 (0.3%) |
| **House context** |  |
| Rural | 93 (25.5%) |
| Urban | 271 (74.5%) |
